# Splice-Switching Therapy for Spinal Muscular Atrophy

**DOI:** 10.3390/genes8060161

**Published:** 2017-06-12

**Authors:** Katharina E. Meijboom, Matthew J.A. Wood, Graham McClorey

**Affiliations:** Department of Physiology, Anatomy and Genetics, University of Oxford, South Parks Road, Oxford OX1 3QX, UK; karin.meijboom@dpag.ox.ac.uk (K.E.M.); matthew.wood@dpag.ox.ac.uk (M.J.A.W.)

**Keywords:** spinal muscular atrophy, splice-switching oligonucleotides, clinical trials

## Abstract

Spinal muscular atrophy (SMA) is a genetic disorder with severity ranging from premature death in infants to restricted motor function in adult life. Despite the genetic cause of this disease being known for over twenty years, only recently has a therapy been approved to treat the most severe form of this disease. Here we discuss the genetic basis of SMA and the subsequent studies that led to the utilization of splice switching oligonucleotides to enhance production of SMN protein, which is absent in patients, through a mechanism of exon inclusion into the mature mRNA. Whilst approval of oligonucleotide-based therapies for SMA should be celebrated, we also discuss some of the limitations of this approach and alternate genetic strategies that are currently underway in clinical trials.

## 1. Introduction

Spinal muscular atrophy (SMA) is an autosomal recessive disorder that occurs in 1:6000–1:10,000 newborns [1] and is the leading genetic cause of infant mortality [2]. The major pathological component of the disease is the selective loss of lower alpha motor neurons in the ventral horn of the spinal cord, resulting in progressive muscle denervation [3], skeletal muscle atrophy and eventually, paralysis and death [4]. SMA can be classified into five types, which are based on severity of clinical symptoms and age of onset (Table 1). The molecular basis of SMA in more than 95% of patients is homozygous deletions or mutations within the *survival motor neuron 1* (*SMN1*) gene [5] and subsequent loss of functional SMN protein. SMN protein is involved in the assembly of small nuclear ribonucleic proteins (snRNP), which underlies its predominant role in splicing regulation [6], although its specific role in SMN pathology is not fully understood. SMN protein is ubiquitously expressed, however motor neurons in the anterior horn of the spinal cord are most severely affected by SMN depletion [7] and restoration of muscle SMN protein alone is not enough for phenotypic improvement [8]. Despite the genetic cause of SMA being known for more than twenty years there has been no effective approved treatment until now. Recent Food and Drug Administration (FDA) approval has been granted for a splice-switching antisense drug for SMA and in this review we outline the preclinical and clinical studies that led to this approval as well as discuss potential strategies to overcome constraints in current treatment possibilities.

## 2. Regulation of Alternative Splicing in Exon 7 of *SMN2* Gene

In humans, a near identical centromeric copy of the *SMN1* gene exists, termed *SMN2*. One critical difference between *SMN2* and *SMN1* is a single nucleotide change (C to T) 6 base pairs into exon 7 [9], which does not alter an amino acid, but results in exon 7 exclusion from ~90% of the mature transcript [10]. This protein, termed SMN∆7, does not efficiently oligomerize, is unstable and gets rapidly degraded. Significantly for affected individuals, ~10% of *SMN2* transcripts will include exon 7 in the mature transcript and thus produce some functional SMN protein that confers survival beyond birth. Furthermore, as the *SMN2* gene is subject to gene duplication, the number of copies, and hence the total level of functional SMN protein, is the main determinant in the clinical outcome for patients with SMA, as outlined in Table 1. However the role of genetic modifiers should also be recognised, with research on differentially affected siblings demonstrating that modifiers such as plastin 3 [11] and neuritin 1 can alter SMA patient phenotype [12]. Exon 7 of the *SMN1/2* gene is characterized as having weak 3′ and 5′ splice sites [13], and its inclusion into the mature transcript is determined by cis-acting elements such as intronic splicing silencers (ISSs), intronic splicing enhancers (ISEs), exonic splicing silencers (ESSs) and exonic splicing enhancers (ESEs) [14]. Alternate hypotheses have been proposed as to how the cytosine to thymine/uracil transition causes exclusion of exon 7 in 90% of mature transcripts. For example some models explain that this transition would abrogate an essential ESE [15] or create new ESSs [16,17] or could strengthen the inhibitory context of the sequence [18].

**Figure 1:**
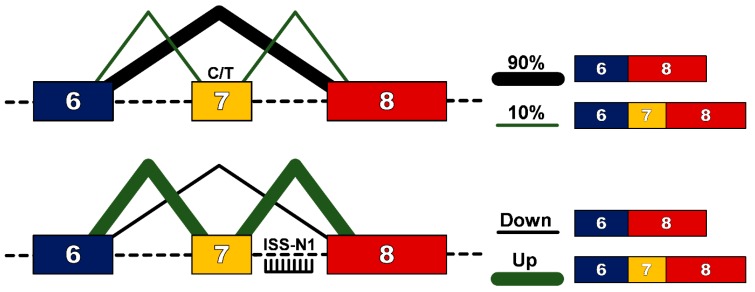
Schematic of splice-switching strategy to redirect alternative splicing pattern in the *SMN2* gene. A C>T transition at position 6 of exon 7 of the *SMN2* gene disrupts an exon splicing enhancer region resulting in exclusion of exon 7 from 90% of the mature SMN transcript. Splice-switching oligonucleotides targeting the intronic splice suppressor N1 (ISS-N1) downstream of exon 7, promotes inclusion of exon 7 and production of a functional SMN protein.

The evidence that even a small increase in the amount of SMN protein can improve clinical outcome makes the ubiquitous *SMN2* gene an attractive genetic target. Furthermore, the presence of “leaky” production of full-length *SMN* transcript suggests that up-regulation of this naturally occurring event could be an attractive strategy for SMA. Modulation of splicing patterns to overcome genetic mutations has been demonstrated in numerous diseases, most notably in Duchenne muscular dystrophy (DMD) [19]. To facilitate this in DMD, splice-switching oligonucleotides (SSO) are designed to bind to pre-mRNA through Watson-Crick base-pairing to either consensus splice sites or splicing enhancer regions so as to prevent recognition of an exon and induce subsequent removal of the exon from the mature transcript so as to restore the reading frame altered by out-of-frame deletion mutations. For SMA however, the challenge is to enhance inclusion, rather than exclusion of exon 7, from the mature transcript. The first such successful attempt was targeting of the 3′ splice site of exon 8, which resulted in an increase in the use of the 3′ splice site of exon 7 and subsequent increase in full-length protein [20]. The decisive breakthrough in this field though came with the identification of a 15 nucleotide sequence splicing silencer element in intron 7, termed intronic splicing silencer-N1 (ISS-N1) [21]. The ISS-N1 sequence is recognized by heterogeneous nuclear ribonucleoprotein A1/A2 (hnRNPA1/A2), a splicing repressor protein. Through specific targeting of this site with SSOs, recognition by hnRNPA1/A2 is blocked, removing the repressor signal and promoting exon 7 inclusion into the mature *SMN2* transcript [22] (Figure 1).

A large body of preclinical studies have been performed in mouse models of SMA to investigate the use of SSOs to target ISS-N1 as a potential therapeutic avenue. Numerous SSO chemistries targeting ISS-N1 have been tested in vivo, including 2′-*O*-methyl phosphorothioate (2′OMePS) [23], phosphorodiamidate morpholino oligomer (PMO) [24,25,26] and 2′-*O*-methoxyethoxy (2′-MOE) [27]. One of the earliest successes was with a 2′MOE chemistry against ISS-N1, named ASO-10–27. Systemic administration to the severe SMA model (*Smn1^−/−^*; *SMN2*) at postnatal day (PND) 0 and 3 of a 160 μg/g/injection of ASO-10-27 resulted in life extension from <12 days to a median survival of 248 days [28]. One crucial aspect of this study was the demonstration that systemic treatment by subcutaneous administration in combination with intracerebroventricular (ICV) administration was much more effective than ICV administration alone (extending the median lifespan by 25-fold), suggesting a synergistic benefit of body-wide SMN restoration. Although significant, this study was performed in PND0/3 mice and thus the potential for SSOs crossing an immature blood brain barrier to contribute to efficacy cannot be ruled out. Indeed in an earlier study, whilst tail vein administration of ASO-10-27 into a mildly affected adult SMA model (*Smn1^+/−^*; *SMN2*) demonstrated enhanced exon 7 inclusion body-wide, no activity was observed in the spinal cord [22], suggesting that direct administration to the central nervous system would be necessary for therapeutic efficacy for this chemistry. The importance of timing of SMN restoration during development of motor neurons and the neuromuscular unit has also been elegantly demonstrated through various studies utilising inducible transgenes [29], virus-mediated *SMN1* replacement [30] and SSO treatment [31] that demonstrate that at least in murine models, the earlier SMN protein restoration is initiated the greater the benefit to survival. 

It should be noted that whilst targeting of ISS-N1 by SSOs has been the most successful strategy to date, other oligonucleotide-based approaches have also been studied. Bi-functional oligonucleotides have been developed that consist of an exon 7 targeting element coupled to a serine-arginine rich protein to enhance recognition and thus promote inclusion by cellular splicing machinery into the mature transcript [32]. This approach was used to successfully demonstrate increased *SMN2* exon 7 inclusion in SMA fibroblasts [33] and ICV injection elicited a robust induction of full length SMN protein in brain and spinal cord in neonatal mice and increased lifespan, motor neuron survival and weight gain in SMN∆7 mice [34]. An alternate approach to *SMN* exon 7 inclusion has been to target a recently identified long non-coding RNA (lncRNA) that is found on the antisense strand of *SMN* (SMN-AS1) [35]. This lncRNA is enriched in neurons and transcriptionally represses SMN expression by recruiting Polycomb repressive complex-2. Combining administration of a 2′MOE gapmer to down-regulate SMN-AS1 with an *SMN2* targeting SSO, increased median survival from 25 days (*SMN2* SSO alone) to 37 days.

## 3. Clinical Trials of SSOs in SMA

The pre-clinical success of ASO-10-27 in severe mouse models of SMA led to IONIS Pharmaceuticals, in conjunction with Biogen, to develop this approach for treatment of SMA patients. Specifically this drug, known variously as ISIS-SMNRx, Nusinersen and SPINRAZA^TM^, is a 18mer SSO comprising 2′-MOE chemistry on a fully modified phosphorothioate backbone. The first clinical trial [36] was initiated in 2011 in a Phase 1 open-label, single-ascending dose study to assess safety, tolerability, pharmacokinetics and clinical effects of intrathecal administration in 28 patients with type 2 and type 3 SMA (age 2–14 years). Both the SSO drug and the lumbar puncture procedure were well tolerated with no adverse effects when treated up to the highest tested single dose of 9 mg. Furthermore, the 9 mg dosing group demonstrated improvement on the Hammersmith Functional Motor Scale Examination (HFMSE) of 17.6% by day 85. Through a further open-label extension study, the majority of children with SMA type 2 and type 3 receiving the two tested highest doses 6 and 9 mg, continued to show improvement in motor function tests with the amelioration of symptoms shown to be dose-dependent and stable [36]. A Phase 2, open-label dose-escalation study assessed the safety, tolerability, pharmacokinetics and clinical efficacy of 6 or 12 mg repeat loading-dose of three times over three months followed by four monthly administration through intrathecal administration in twenty type 1 patients where treatment was initiated between 3 weeks and 6 months upon identification of SMA symptoms. Significantly in this study, repeat intrathecal administration of the drug was well tolerated with no safety concerns identified. Promising improvements in motor milestones and motor functions, survival or permanent ventilation independence and improvements in neuromuscular electrophysiology were observed in most, but not all treated infants [37]. Additionally, analysis of post-mortem tissue indicated that Nusinersen was broadly distributed throughout the spinal cord and brain including the target motor neurons [37,38]. As no safety concerns were identified in this study, a large phase 3 randomised, double-blind, sham-controlled was initiated in 2014 incorporating 122 SMA type 1 patients (ENDEAR), and 126 non-ambulatory type 2 patients (CHERISH) administered 12 mg intrathecal dosing. In August 2016, ENDEAR met its primary endpoints, which were improvements in event-free survival of type 1 patients and increase in the proportion of motor milestone responders in the Hammersmith Infant Neurological Examination; and in November 2016, CHERISH met its primary endpoint, which was a significant positive change in HFMSE scores. Following 15 months treatment, patients in the CHERISH study had achieved an improvement of 4 points in the HFMSE scale compared to a decline of 1.9 points in the sham placebo arm. A similar trend was observed in the ENDEAR study with infants demonstrating a significant improvement in the achievements of motor milestones compared to natural history. The treatment showed again a favourable safety profile and most adverse events were either related to the disease, common events in the general public or because of the lumbar puncture procedure [39]. The evidence from the ENDEAR trial that some of the type I SMA infants treated for three years were surviving without ventilation; and could also have limited ambulation is unprecedented for type 1 SMA patients, since they live normally less than two years and often never achieve the ability to sit, let alone walk. Based on the positive data from this phase 3 trial, in December 2016, the U.S. FDA approved SPINRAZA^TM^ (Nusinersen) as the first and only genetic treatment to date for SMA [40]. Ionis and Biogen have also filed their NDA with the European Medicines Agency, which is currently pending.

## 4. Challenges for SSOs for the Treatment of SMA

There is no doubt that the success of SPINRAZA^TM^ offers great hope to SMA patients and their families to prolong life and improve motor function. However, there are some limitations of the current clinical approach of intrathecal administration by lumbar puncture. Besides being a relatively labour intensive and invasive route of administration, nearly a third of patients experience injection related adverse side effects [41]. Additionally, distribution of the SSO by this route is predominantly limited to the central nervous system that whilst crucial for SMN restoration in motor neurons, may not address the cardiovascular [42], metabolic, liver, pancreatic, intestinal and lung defects observed in SMA patients [43]. This may suggest that an optimal treatment strategy would incorporate SMN protein restoration in both the central nervous system (CNS) and peripheral tissues. Indeed, preclinical mouse studies combining both subcutaneous and direct administration of SSOs has been shown to be much more effective than either strategy alone to promote survival and motor function [28,44]. One of the limitations of systemic administration of SPINRAZA^TM^, and indeed all SSO chemistries studied to date for SMA, is their inherently poor ability to cross into tissue from circulation, especially into the central nervous system, which is tightly controlled by the blood-brain barrier [45,46]. To address this problem in the field of oligonucleotide therapy, a number of delivery platforms have been developed to enhance tissue biodistribution, with the greatest success being targeted delivery to the liver utilising lipid nanoparticles (reviewed here [47]). One of the most promising approaches for SSOs is the use of cell-penetrating peptides (CPPs) that when conjugated to neutral SSO chemistries such as PMOs have been demonstrated to enhance body-wide delivery following intravenous administration [48]. This includes several studies that have demonstrated the use of CPPs to deliver oligonucleotides to the CNS whereby systemic IV delivery of an arginine-rich cell-penetrating peptide conjugated to a PMO, resulted in uptake in all areas of the brain in mice [49]. We have recently demonstrated the potential for CPP-PMO treatment of SMA in a study utilising low-dose intravenous administration in the severe SMA mouse model [24]. Survival of the *SMN1^−/−^*; *SMN2* mouse model was extended from 12 days to a median of 457 days following 2 × 10 µg/g administration of CPP-PMO concurrent with improvements in motor function and neuromuscular junction morphology. Importantly, an increase in exon 7 inclusion and SMN protein levels could be detected in both central nervous system and peripheral muscles. The addition of the CPP was crucial for this observed benefit as naked PMO IV administration at the same dose increased survival to only 54 days. Alternate CNS-targeting CPP designs have also been assessed in SMA with systemic administration of Apolipoprotein E conjugated PMO into *Smn1^−/−^*; *SMN2* mice improving median survival of 10.5 to 78 days [50]. In the same study, intravenous tail vein administration to mildly affected adult SMA mice (*Smn1^+/−^*; *SMN2*) also resulted in significant increase of full length *SMN2* in the CNS and peripheral tissues. Whilst CPP based delivery systems have demonstrated potential in both SMA and DMD animal studies the underlying issue of drug toxicity, whereby liver and kidney damage has been demonstrated following high dose administration of CPPs [51,52] remains to be addressed. If this problem can be overcome through further iterations of CPP design, then this will be an attractive approach for body-wide induction of SMN protein.

## 5. Non-SSO Strategies SMN Up-Regulation

Whilst SPINRAZA^TM^ is the first approved treatment for SMA, other strategies to upregulate expression of SMN protein are under clinical investigation. The use of viral-mediated delivery of the *SMN1* gene has demonstrated extension of lifespan in SMA mice [53,54] and porcine [55] models. In 2014, AveXis initiated a Phase 1 open-label, dose-escalation trial, testing the safety and efficacy of intravenous delivery of AAV9-SMN (AVXS-101) in SMA type 1 patients. Interim Phase I data from a cohort of twelve patients receiving the proposed therapeutic dose demonstrated that AVXS-101 was generally well tolerated. All patients reached 13.6 months of age event-free as defined by death or requiring at least 16 h per day ventilation, whereas the event-free survival rate based on natural history is 25%. A mean increase in the baseline of The Children’s Hospital of Philadelphia Infant Test of Neuromuscular Disorders (CHOP-INTEND) [56] test of 24.7 points was also observed in this cohort reflecting improvements in motor function with 2/12 patients achieving scores considered in the range of normal with 5/12 patients able to sit unassisted for more than 30 s [57]. Despite this success, a potential drawback of systemic viral delivery of an *SMN1* transgene is the activation of the immune system [58], which could limit the possibility of multiple treatments. However, considering the importance of SMN protein in development it may be that significant restoration of SMN protein at an early stage through viral delivery could have a significant benefit to clinical prognosis. Furthermore, administration of a virally-delivered transgene should not preclude further treatment with SSO based therapies at a later date.

Histone deacetylase (HDAC) inhibitors have also been used to promote *SMN2* transcription to increase levels of full-length SMN protein. Valproic acid (VPA), which has been used in the clinic to treat epilepsy, had been shown to increase SMN protein levels in SMA fibroblasts [59]. Treatment with VPA in phase 2 open label studies demonstrated increased muscle strength and motor function in type 2 [60] and type 3/4 patients [61]. However, in later clinical trials with SMA type 3 and 4 patients receiving similar doses, no beneficial effect was demonstrated after 12 months treatment [62,63]. Inconsistency in these clinical results has been suggested to be due to responders and non-responders to the drug, suggesting pre-selection of potential responders could improve the potential for this approach [64].

## 6. Conclusions

Spinal muscular atrophy is the leading genetic cause of infant mortality and finally, after the discovery of the causative gene 21 years ago, the first ever treatment for SMA has been approved. Whilst this approval is great news for parents and patients alike, it should be tempered by this approach representing a treatment and not a cure. Although clinical trial data does show an improvement for many patients there are still non-responders and improvement in clinical outcomes is varied. Additionally, whilst SPINRAZA has been determined to be safe, an intrathecal administration route by lumbar puncture is less than ideal. Administration through a more systemic route would be more desirable and likely better address the peripheral requirements for SMN protein, although it must be recognised that the role for SMN protein in central nervous development is the most crucial aspect of the disease. Further development of alternate genetic approaches including cell-penetrating SSOs and viral transgene delivery could be expected to complement, if not improve upon SPINRAZA treatment, and indeed it could be foreseen that a combination therapy of early intervention to the CNS followed by peripheral restoration of SMN protein may be the optimal genetic strategy. The importance of therapies that address underlying disease pathology such as peripheral or motor function defects should also continue to be investigated as whilst alone they would not be expected to prevent disease progression, they could be expected to have a synergistic benefit with SMN protein restoration, especially in type III/IV patients who already have low levels of SMN protein. Regardless of the limitations of SPINRAZA, its approval, along with Eteplirsen for Duchenne muscular dystrophy, have been important for the antisense therapeutic field and should provide an impetus for further oligonucleotide-based drugs to be investigated for genetic disorders. More importantly though, for patients and especially for families of infants with type I SMA, this approach provides the first step towards a significant improvement in the quality and extension of life.

## Figures and Tables

**Table 1:** Relationship of *SMN2* copy number to disease classification, age of onset, typical symptoms and life expectancy.

Type	Age of Onset	Symptoms	Life Expectancy	*SMN2* Copies
0	Prenatal	Less active foetus	<birth	1
1	0–6 months	Cannot sit, respiratory muscle weakness	<2 years	2
2	6–18 months	Cannot walk	<40 years	3, 4
3	18 months–5 years	Needs support to walk	Adult	3, 4
4	>5 years	Restricted mobility	Adult	4–8
